# Improving predictive ability in sparse testing designs in soybean populations

**DOI:** 10.3389/fgene.2023.1269255

**Published:** 2023-11-23

**Authors:** Reyna Persa, Caio Canella Vieira, Esteban Rios, Valerio Hoyos-Villegas, Carlos D. Messina, Daniel Runcie, Diego Jarquin

**Affiliations:** ^1^ Agronomy Department, University of Florida, Gainesville, FL, United States; ^2^ Crop, Soil, and Environmental Sciences, Bumpers College, University of Arkansas, Fayetteville, AR, United States; ^3^ Department of Plant Science, McGill University, Montreal, QC, Canada; ^4^ Horticultural Sciences Department, University of Florida, Gainesville, FL, United States; ^5^ Department of Plant Sciences, University of California Davis, Davis, CA, United States

**Keywords:** sparse testing, genomic prediction, plant breeding, soybean, experimental design, genotype-by-environment interaction

## Abstract

The availability of high-dimensional genomic data and advancements in genome-based prediction models (GP) have revolutionized and contributed to accelerated genetic gains in soybean breeding programs. GP-based sparse testing is a promising concept that allows increasing the testing capacity of genotypes in environments, of genotypes or environments at a fixed cost, or a substantial reduction of costs at a fixed testing capacity. This study represents the first attempt to implement GP-based sparse testing in soybeans by evaluating different training set compositions going from non-overlapped RILs until almost the other extreme of having same set of genotypes observed across environments for different training set sizes. A total of 1,755 recombinant inbred lines (RILs) tested in nine environments were used in this study. RILs were derived from 39 bi-parental populations of the Soybean Nested Association Mapping (NAM) project. The predictive abilities of various models and training set sizes and compositions were investigated. Training compositions included a range of ratios of overlapping (O-RILs) and non-overlapping (NO-RILs) RILs across environments, as well as a methodology to maximize or minimize the genetic diversity in a fixed-size sample. Reducing the training set size compromised predictive ability in most training set compositions. Overall, maximizing the genetic diversity within the training set and the inclusion of O-RILs increased prediction accuracy given a fixed training set size; however, the most complex model was less affected by these factors. More testing environments in the early stages of the breeding pipeline can provide a more comprehensive assessment of genotype stability and adaptation which are fundamental for the precise selection of superior genotypes adapted to a wide range of environments.

## 1 Introduction

Soybean [*Glycine max* (L.) Merr.] delivers the highest amount of protein per hectare than any crop and accounts for over 60% of total global oilseed production (United States Department of Agriculture, 2022a). It is the largest and most concentrated segment of global agricultural trade and one of the most essential crops to the world’s food security (Gale et al., 2019). Soybean production has nearly doubled over the last two decades (182,830 to 363,860 MT) (United States Department of Agriculture, 2022b). The genetic improvement of soybean cultivars, as well as advancements in farming technology and agronomic practices, have significantly contributed to this substantial increase (Specht et al., 1999; Rowntree et al., 2013; Koester et al., 2014; Rincker et al., 2014; Canella Vieira and Chen, 2021). A typical soybean breeding pipeline consists of several years of multi-environment field trials to select and advance high-yielding breeding lines (Canella Vieira and Chen, 2021; Yoosefzadeh-Najafabadi and Rajcan, 2022). The availability of high-dimensional genomic data (Song et al., 2013; 2020) and the advancements in genome-based prediction models (GP) have revolutionized and contributed to accelerated genetic gains as well as higher testing efficiency in soybean breeding programs (Jarquin et al., 2014a; Jarquin et al., 2014b; Persa et al., 2020; Widener et al., 2021; Canella Vieira et al., 2022).

The concept of GP revolves around using the information of all molecular markers—regardless of estimated effect size of significance—to develop prediction models of the genetic merit for the phenotype of interest in unobserved genotypes (Meuwissen et al., 2001). Thus, GP allows the identification and selection of desirable genotypes earlier in the breeding pipeline, which not only reduce cost, time, and space but enhance genetic gain by shortening the length of the breeding cycle and increasing selection intensity (Jarquin et al., 2014a; Crossa et al., 2017; Canella Vieira and Chen, 2021; Wartha and Lorenz, 2021; Canella Vieira et al., 2022). However, the presence of the genotype-by-environment G×E interaction, a change in the response patterns from one environment to another, complicates the selection of improved cultivars (Crossa et al., 2011). For this reason, it is necessary to establish multi-environment trials (METs) to evaluate the performance of genotypes under a wide range of weather conditions (environmental stimuli) allowing the selection of stable materials or materials with local adaptation only (Jarquin et al., 2020a). As expected, the high phenotyping cost does not permit the evaluation of all candidate genotypes in all of the environments of interest but a fraction of these combinations of genotypes-in-environments (Jarquin et al., 2020b). To overcome these disadvantages (i.e., G×E and the high phenotyping costs), the implementation of the reaction norm model (Jarquin et al., 2014a) leverages the borrowing of information of genotypes across environments helping to increase the predictability of unobserved combinations of genotypes-in-environments.

In addition, GP including G×E model parameters can substantially improve field testing design and efficiency, as well as resource allocation (Jarquin et al., 2020b; Montesinos Lopez et al., 2023a). For instance, GP can reduce the costs and space associated with field testing by using sparse testing designs in which only a subset of the genotypes are tested at each location (Jarquin et al., 2020b). Sparse testing allows the prediction of non-observed genotype-in-environment combinations reducing the costs at a fixed evaluation capacity (less expensive to make accurate inferences on the original set of genotypes-in-environment combinations) or increasing the overall evaluation capacity at fixed costs (inferences on more genotypes and environment combinations based on the original budget) (Jarquin et al., 2020b).

Using two maize (*Zea mays* L.) data sets from the International Maize and Wheat Improvement Center (CIMMYT)’s breeding program in eastern Africa, Jarquin et al. (2020b) were the first to demonstrate that GP models could substantially reduce the testing footprint of breeding programs using sparse testing designs. Additional studies of GP-based sparse testing designs have been reported in wheat (*Triticum* L.) (Crespo-Herrera et al., 2021; He et al., 2021; Atanda et al., 2022; Montesinos Lopez et al., 2023a; Montesinos Lopez et al., 2023b), maize (Montesinos Lopez et al., 2023b), groundnut (*Arachis hypogaea* L.) (Montesinos Lopez et al., 2023a), and rice (*Oryza sativa* L.) (He et al., 2021; Montesinos Lopez et al., 2023b). To date, no applications of GP-based sparse testing have been reported in soybean. Therefore, the objective of this study is to investigate the potential of reducing the field testing footprint (less natural resources such as land and water associated with the in fields evaluation of RILs) in soybean breeding programs based on sparse testing designs, as well as the prediction accuracy derived from different GP models including the main effect of the molecular markers via covariance structures (M1), a multiplicative reaction norm model (M2) to account for the genotype-by-environment G×E interaction, and an extended reaction norm model also including the family structure (M3) in interaction with environmental stimuli (Persa et al., 2020). Two different methods for model calibration were considered. The first one, is initially based on RILs randomly selected then varying sample sizes and training composition (between non-overlapping [NO-RILs] and overlapping [O-RILs] genotypes across environments) for a fixed testing set size. While the second one, only varies the training set size since it is based on common sets of genotypes observed across environments and selected under a genetic criteria/algorithm, where the goal is to select a core sample of RILs that maximizes/minimizes the genomic diversity on a sample of fixed size. The objectives of implementing this second selection method were to assess the impacts in predictive ability using different levels of genomic diversity of the RILs when calibrating models, and evaluate the stability of these selected RILs across environments. The impacts in predictive ability using these selection methods were evaluated using a soybean population of 1,755 genotypes evaluated in nine environments (all genotypes in all environments).

## 2 Materials and methods

### 2.1 SoyNAM dataset

Phenotypic and genomic data from the Soybean Nested Association Mapping (SoyNAM) experiment (https://www.soybase.org/SoyNAM/) were used in this study. Briefly, the SoyNAM data is comprised of 5,600 recombinant inbred lines (RILs) derived from 40 bi-parental populations (140 RILs per population) corresponding to 40 founders belonging to three different genetic backgrounds [G1: 17 high-yielding lines, G2: 15 diverse ancestries, and G3: eight exotic plant introductions (PI)] crossed with a common hub parent (IA3023) (Diers et al., 2018). The common parent and founder lines, the RILs and check cultivars were grown in two-row field plots (0.76 m spacing; *ca*. 4 m long) and phenotyped for nine agronomic traits including grain yield (kg ha^-1^), plant height (cm), seed protein and oil (% dry weight), days to maturity (number of days from planting when 95% of the plant reach physiological maturity), seed size (100 seeds weight in grams), fiber content (percentage in the grain), lodging (score from 1–5), and shattering (score from 1 to 5). Initially, the RILs from each family were split in four sets of 35 and each set was augmented with the two parents of the family and three check cultivars selected for adaptation to the field environment; however, if there was not enough seed available for a RIL, the plot was completed with a check variety. RILs were genotyped using the Illumina Infinium BARCSoySNP6K BeadChip (Song et al., 2020). After filtering molecular markers with more than 20% of missing values and minor allele frequency smaller than 0.03, a subset of 4,100 single nucleotide polymorphisms (SNPs) was available for data analysis.

Balanced multi-environment field experiments where all genotypes are observed in all testing environments are essential to assess the efficacy and advantages of the sparse testing design. For this, environments with less than 1,500 overlapping RILs across all testing environments were discarded. After applying this criterion to the phenotypic data, a total of 1,775 RILs derived from 39 bi-parental families (16 G1, 15 G2, and eight G3) remained for analyses (all RILs tested in all nine environments). The nine environments considered in this study were located across five States (Iowa, Illinois, Indiana, Kansas, and Nebraska) in 2012 and 2013. These included Iowa 2012 (IA_2012), Iowa 2013 (IA_2013), Illinois 2012 (IL_2012), Illinois 2013 (IL_2013), Indiana 2012 (IN_2012), Indiana 2013 (IN_2013), Kansas 2012 (KS_2012), Kansas 2013 (KS_2013), and Nebraska 2012 (NE_2012).

### 2.2 Training set selection and composition methods

Two different selection methods were considered to compose calibration sets. The first method (S1) is based on randomly selecting (for each replicate) sets of 195 RILs (1,755 divided by nine) and assigning non-overlapped to each one of the nine environments (total of five replicates). In this case, the 1,755 phenotypic observations measured across nine environments correspond to roughly 11% of all the total potential RILs-in-environment combinations (1,755 × nine environments = 15,795). The objective is to predict the remaining 1,560 non-observed RILs (1,755–195) in each environment for a total of 14,040 (1,560 × nine environments) missing combinations across all environments. In addition, different training set sizes were considered by systematically reducing the training set size by groups of 10 RILs from 195 to 95 RILs within each environment. For instance, by reducing the initial training set size (195) by 10 RILs, the training set size across environments was reduced to 1,665 (1,755–90). By reducing the training set by 100 RILs, the training set size across environments was reduced to 855 (1,755–900). The different within environments training set sizes varied from 195 to 95 RILs (or equivalently from 1,755 to 855 across environments).

**FIGURE 1:**
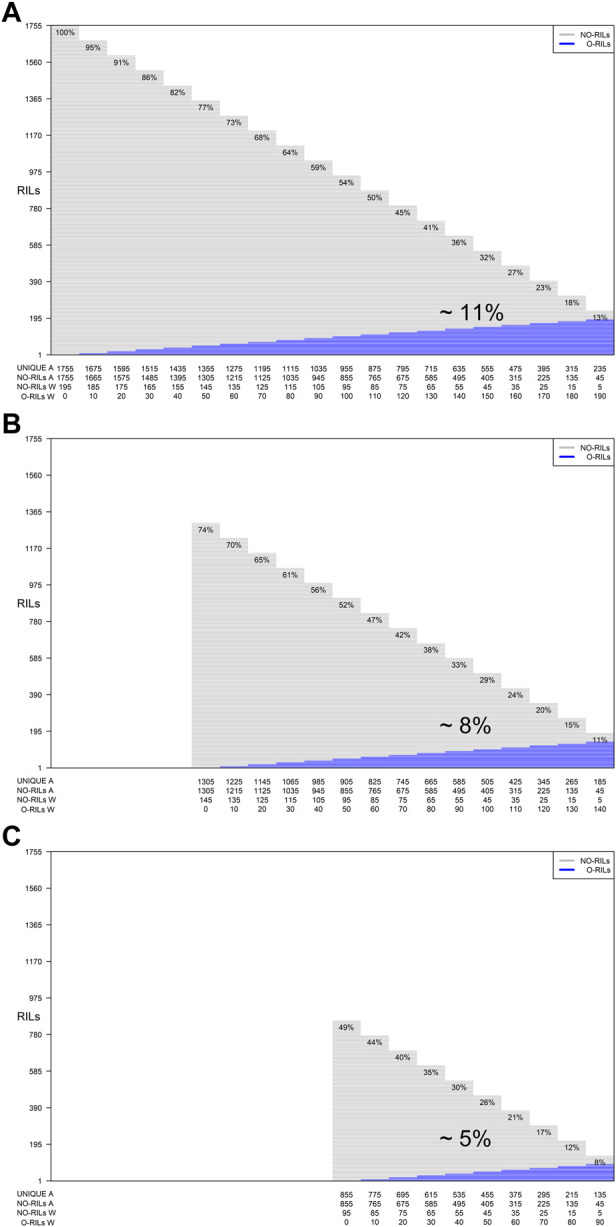
Graphical representation of three different training set sizes, compositions and genetic diversity based on the number of unique RILs used for model calibration. The horizontal gray lines correspond to NO-RILs (A across/W within environments) while the horizontal blue lines represent O-RILs; values in the diagonal provide information about the percentage of unique RILs (UNIQUE A) across environments. **(A)** corresponds to the selection of 195 RILs per environment for a total of 1,775 phenotypic observations. **(B, C)** correspond to intermediate and a reduced training set sizes with 145 and 95 RILs per environment with different compositions.

In addition, for each of the training set sizes (195, 185, 175, 165, 155, 145, 135, 125, 115, 105, and 95), different training compositions consisting of non-overlapping (NO-RILs) and overlapping (O-RILs) genotypes across environments were considered under the S1 selection method (Table 1). To compose these, the starting point were the original sets of 195 NO-RILs that were assigned to each environment. Then, within each environment, 10 RILs were masked as non-observed reducing the total number to 185 NO-RILs. For the within environments training set size of 195 NO-RILs, after masking 10 RILs as non-observed the total number of NO-RILs is reduced to 185. Out of the 90 RILs masked as non-observed (10 non-observed RILs × nine environments), 10 were randomly selected as O-RILs to be observed across all environments. The total number of 195 RILs was consistently observed within each environment such that 185 were NO-RILs and 10 were O-RILs (Table 1). Thus, the total number of unique tested RILs across environments was reduced from 1,755 to 1,675 (185 NO-RILs × nine environments +10 O-RILs) and the number of NO-RILs across environments was reduced to 1,665 (185 NO-RILs × nine environments). The removal of 10 NO-RILs within environments and redistribution as 10 O-RILs across environments were conducted systematically until reaching the five NO-RILs and 190 O-RILs composition within environments (Figure 1). In such composition, the total number of testing plots remained at 1,755 (195 RILs × nine environments), but the total number of unique RILs across environments was reduced to 235 (five NO-RILs × nine environments +190 O-RILs), and the number of NO-RILs across environments was reduced to 45 (five NO-RILs × nine environments) (Figure 1). The removal of 10 NO-RILs within environments and redistribution as 10 O-RILs across environments was conducted systematically in each training set size ranging from 195 to 95 RILs per environment (Table 1).

**TABLE 1:** Summary of different training set sizes and compositions for selection method S1.

RILs	Training set composition (non-overlapping RILs - overlapping RILs)																			
195	195-0	185-10	175-20	165-30	155-40	145-50	135-60	125-70	115-80	105-90	95-100	85-110	75-120	65-130	55-140	45-150	35-160	25-170	15-180	5-190
185	-	185-0	175-10	165-20	155-30	145-40	135-50	125-60	115-70	105-80	95-90	85-100	75-110	65-120	55-130	45-140	35-150	25-160	15-170	5-180
175	-	-	175-0	165-10	155-20	145-30	135-40	125-50	115-60	105-70	95-80	85-90	75-100	65-110	55-120	45-130	35-140	25-150	15-160	5-170
165	-	-	-	165-0	155-10	145-20	135-30	125-40	115-50	105-60	95-70	85-80	75-90	65-100	55-110	45-120	35-130	25-140	15-150	5-160
155	-	-	-	-	155-0	145-10	135-20	125-30	115-40	105-50	95-60	85-70	75-80	65-90	55-100	45-110	35-120	25-130	15-140	5-150
145	-	-	-	-	-	145-0	135-10	125-20	115-30	105-40	95-50	85-60	75-70	65-80	55-90	45-100	35-110	25-120	15-130	5-140
135	-	-	-	-	-	-	135-0	125-10	115-20	105-30	95-40	85-50	75-60	65-70	55-80	45-90	35-100	25-110	15-120	5-130
125	-	-	-	-	-	-	-	125-0	115-10	105-20	95-30	85-40	75-50	65-60	55-70	45-80	35-90	25-100	15-110	5-120
115	-	-	-	-	-	-	-	-	115-0	105-10	95-20	85-30	75-40	65-50	55-60	45-70	35-80	25-90	15-100	5-110
105	-	-	-	-	-	-	-	-	-	105-0	95-10	85-20	75-30	65-40	55-50	45-60	35-70	25-80	15-90	5-100
95	-	-	-	-	-	-	-	-	-	-	95-0	85-10	75-20	65-30	55-40	45-50	35-60	25-70	15-80	5-90

The second method (S2) implemented to select RILs and compose training sets is an alternative to the ramdom method and it is based on the genetic diversity of the RILs using genomic information. It focuses mainly on the maximization/minimization of the genetic diversity. For this, the Super Saturated Design (SSD) method was implement to select samples of fixed size (selected from a large pool of genotypes) increasing (SSD.max) or decreasing (SSD.min) the genetic diversity in the sample (Virdi et al., 2023). Here, the initial fixed training set size for model calibration consisted of 195 O-RILs across environments. Since the selection of RILs does not involve a random process, for each case (maximize/minimize genetic diversity) only one sample was obtained. Repeating the selection algorithm using a random sample of 195 RILs as starting point would return nearly identical samples (>98%). Briefly, the genomic information of the 1,755 RILs was randomly split into two independent sets, one of size 195 ( $X_{0}$ ) and the other of size 1,560 ( $X_{1}$ ). The first set $X_{0}$ was used to store the selected RILs that met the criteria of maximizing/minimizing the genetic diversity of the sample while the RILs in $X_{1}$ were candidates to be selected to compose the sample of size 195.

Systematically, for each iteration, each RIL (one at a time) from the selected set of 195 RILs $\left(X_{0}\right.$ ) ( i=1,2,..,195 ) is replaced by each one of the 1,560 RILs (*j* = 1,2, … , 1,560) from the $X_{1}$ set. Then, *E*(*S* ^2^) is computed, where $S=X_{0}^{\prime }X_{0}$ is a matrix of genetic similarities between pairs of markers for a given group of individuals, $X_{0}$ is the SNPs matrix of dimension 195 × 4,100. The $E\left(S^{2}\right)$ is defined as the sum of the squared values of the off-diagonal elements of $S^{2}$ . Equivalently, maximizing/minimizing the $E\left(S^{2}\right)$ can be accomplished by respectively increasing/decreasing the trace (sum of the values in the diagonal) of $S^{2}$ . Thus, for one iteration, the total number of times that the *E*(*S* ^2^) is computed is 304,200 (195 ×1,560) and the objective is to identify the *l* ^th^ (*j* = 1, 2, … , 1,560) RIL in the $X_{1}$ set that maximizes or minimizes (according to the desired sample) the $E\left(S^{2}\right)$ after discarding the *m* ^th^ (*i* = 1, 2, … , 195) RIL of the $X_{0}$ set. Thus, out of the 304,200 combinations, only one satisfies the condition of maximizing/minimizing the most the $E\left(S^{2}\right)$ . These two RILs are exchanged from one set to the other (i.e., substitute the *l* ^th^ RIL in $X_{0}$ by the *m* ^th^ RIL in $X_{1}$ ). This procedure is repeated until $E_{\min}\left(S_{l,m}^{2\left(k\right)}\right)\leq E_{\min}\left(S_{l,m}^{2\left(k+1\right)}\right)$ for the *k* ^th^ iteration $\left(k=1,2,\ldots \right)$ when the objective is to maximize the genetic diversity in the sample or $E_{\max}\left(S_{l,m}^{2\left(k\right)}\right)\geq E_{\max}\left(S_{l,m}^{2\left(k+1\right)}\right)$ to minimize it.

### 2.3 Genomic prediction models

#### 2.3.1 M1: E + G; environment and genomic main effects

Consider that $y_{ij}$ represents the yield performance of the *i* ^th^ genotype at the *j* ^th^ environment, and it is composed of the sum of a common effect (μ) plus an environmental random effect ( $E_{j}$ , *j* = 1,2, … , *J*), a genetic random effect corresponding to the *i* ^th^ RIL ( $L_{i}$ , *j* = 1,2, … , *I*), a genomic random effect ( $g_{i}$ , *i* = 1,2, … , *I*), and a random error term $\left(\epsilon_{ij}\right)$ capturing the unexplained variability by the model components. This linear predictor can be written as follows:

$$y_{ij}=\mu +E_{j}+L_{i}+g_{i}+\epsilon_{ij}$$ (1)

where $E_{j}∼N\left(0,\sigma_{E}^{2}\right)$ and $\sigma_{E}^{2}$ represents the corresponding variance component; $g=\left\{g_{i}\right\}∼N\left(0,{G\sigma }_{g}^{2}\right)$ , $G=XX^{\prime }/p$ , **X** is the centered and standardized (by columns) matrix of SNPs, $\sigma_{g}^{2}$ is the additive genetic variance; and $\epsilon_{ij}∼N\left(0,\sigma^{2}\right)$ with $\sigma^{2}$ as the residual variance. The entries of G describe the genomic similarities between pairs of individuals allowing the borrowing of information between tested and untested genotypes.

#### 2.3.2 M2: E + G + GE; environment and genomic main effects plus genotype-by-environment (GE) interaction

The previous M1 returns a singular genomic effect for each genotype observed in different environments. Thus, the resulting predicted genomic effect might not be accurate when considering all tested environments. To allow specific genomic effects at each environment, a model incorporating the genotype-by-environment GE interaction was considered. The reaction norm model conceptually allows the inclusion of the interaction between each molecular marker and each environmental factor in a convenient way via covariance structures (Jarquin et al., 2014a). Let $\left.{\text{gE}=\{\text{gE}}_{\text{ij}}\right\}$ be the vector of the interaction scores between *i* ^th^ genotype and the *j* ^th^ environment. The previous interaction effect can be modeled as a random effect following a multivariate normal distribution centered on zero and a covariance structure given by $Z_{g}GZ_{g}^{\prime }\circ Z_{E}Z_{E}^{\prime }$ where $Z_{g}$ and $Z_{E}$ represent the incidence matrices that connect phenotypic records with genotypes and environments, respectively. Here, “ ∘ ” is the Hadamard product (cell-by-cell) between two matrices (covariances structures). Adding the previous model term to M1, the resulting linear predictor is as follows:

$$y_{ij}=\mu +E_{j}+L_{i}+g_{i}+{\text{gE}}_{ij}+\epsilon_{ij}$$ (2)

where ${gE=\{\text{gE}}_{ij}\}∼N\left(0,Z_{g}GZ_{g}^{\prime }\circ Z_{E}Z_{E}^{\prime }\sigma_{gE}^{2}\right)$ with $\sigma_{gE}^{2}$ representing the associated variance component.

##### 2.3.3 M3: E + G + GE + FE; Environment and Genomic main effects plus genotype-by-environment and family-by-environment interactions

Persa et al. (2020) proposed a model to leverage the information of individuals belonging to the same families but observed in different environments by including the family-by-environment FE interaction model term. Consider $F_{k}\;\left(k=1,2,\ldots ,m\right)$ , as the random model term representing the effect of the *k* ^th^ family such that $F_{k}∼N\left(0,\sigma_{F}^{2}\right)$ . This model term allows the borrowing of information between genotypes belonging to the same family under the premise that genotypes from the same family may perform alike. Here, the predicted effect of the family membership is common for individuals of the same family but observed in different environments. For this reason, similarly to model M2, the interaction between this model term and the environments $\left.{\text{FE}=\{\text{FE}}_{\text{kj}}\right\}$ was considered to allow specific values for each family at each environment besides the main effect of the family membership. The resulting model after adding the main effect of the family F and the interaction FE to model M2 is as follows:

$$y_{ij}=\mu +E_{j}+L_{i}+F_{k}+g_{i}+{\text{gE}}_{ij}+{\text{FE}}_{kj}+\epsilon_{ij}$$ (3)

where ${FE=\{\text{FE}}_{kj}\}∼N\left(0,Z_{F}Z_{F}^{\prime }\circ Z_{E}Z_{E}^{\prime }\sigma_{\text{FE}}^{2}\right)$ , $\sigma_{\text{FE}}^{2}$ is the corresponding variance component, and $Z_{F}$ is the incidence matrix that connects phenotypes with families.

### 2.4 Assessment of model efficiency

Predictive ability was measured on a trial basis, thus the Pearson correlation coefficient between predicted and observed values was computed within environments. The overall predictive ability was computed as the average Pearson’s correlation coefficient across the nine environments. As mentioned before, despite the training set size or its composition (NO-RILs and O-RILs), the testing set size (prediction set) was the same for all cases. Within each environment, between 95 and 195 RILs were used as the training set for a constant prediction set size of 1,560 RILs.

Since the RILs belong to families with different genetic backgrounds (G1, G2, and G3), the Pearson’s correlation coefficient was also calculated considering only the RILs within each one of the different groups at each environment. In this case, in each environment, the Pearson’s correlation coefficient between predicted and observed values was computed three times, one for each genetic background group. The objective of considering the different groups of families when computing the correlations was to assess the effects on predictive ability when blocking for population structure although that was not the main goal of this research.

## 3 Results

### 3.1 Phenotypic data and population structure

Phenotypic information on grain yield was available for 1,755 genotypes observed in nine environments (all genotypes in all environments) for a total of 15,795 records available for analysis. No statistically significant differences were observed for yield across the seven different initial samples of size 195 RILs selected with the S1 (five replications based on random selections) or S2 (two for maximizing/minimizing the genetic diversity) selection methods. However, the sampling method minimizing genetic diversity resulted in higher average yield (3,722.5 kg ha-^1^) compared to the average of all other sampling methods of (3,563.2 kg ha-^1^) and a significantly lower coefficient of variation among samples (Supplementary Figure S1).

To assess the stability across environments of RILs selected with the different sampling methods, the Pearson’s correlation coefficient between environments was computed for each sample considering the largest sample size (195 RILs) observed across all nine environments. The total number of correlation values among the nine environments was 36 $(\frac{9\times 8}{2})$ . The SSD.max sample showed the highest (0.292) median Pearson’s correlation coefficient with a larger dispersion between the 50% and 75% quantiles while the SSD.min returned the lowest median correlation (0.123) (Supplementary Figure S2).

**FIGURE 2:**
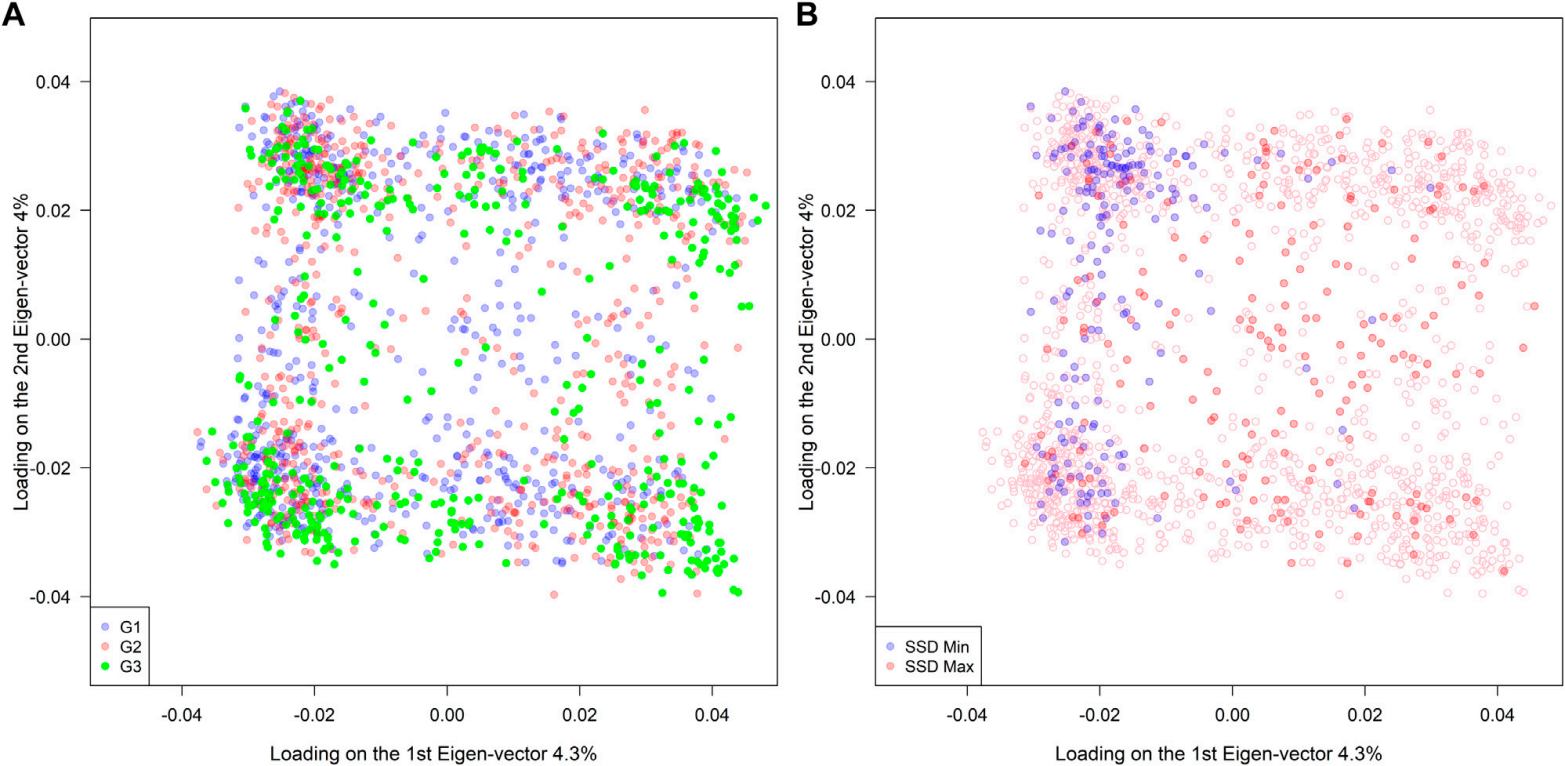
Population structure of 1,755 RILs derived from 39 families sharing a common hub parent (IA3023). The families belong to three different groups of ancestry G1(elite), G2 (diverse), and G3 (exotic background). The right panel indicates the selected genotypes using the super saturated design (SSD) method to maximize (red color) or minimize (blue color) the genetic diversity based on genomic information.

A total of 4,100 SNPs were included in the analysis after filtering the original set of 5,400 SNPs. Among the 1,755 RILs, no clear association patterns regarding population structure were observed across different genetic background groups (G1: high-yielding lines, G2: diverse ancestries, and G3: exotic PIs) (left panel in Figure 2). On the other hand, the SSD.min samples were substantially more clustered than the SSD.max samples when compared to the distribution of all RILs (right panel in Figure 2). This indicates that the SSD methodology can effectively select a group of individuals to either maximize or minimize genetic diversity given a fixed sample size.

### 3.2 Predictive ability across multiple samples

#### 3.2.1 Across ancestry groups

**FIGURE 3:**
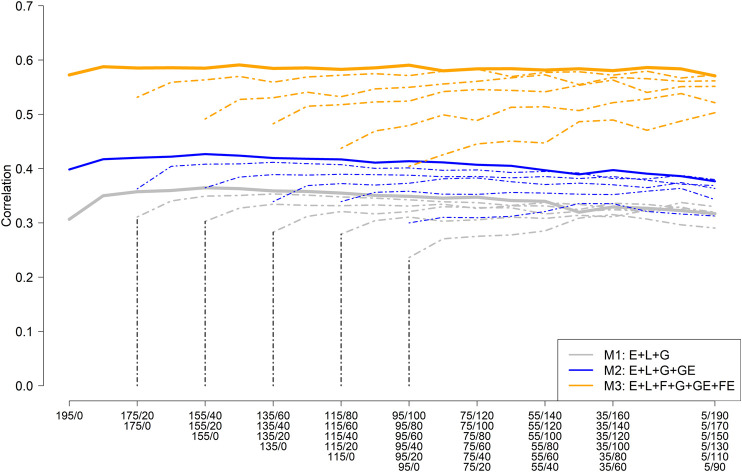
Mean (five replicates) average (across nine environments) of the within environments correlation between predicted and observed values for different sample sizes and composition for model training and three prediction models including the main effect of the environment (E), RIL (L), marker SNPs (G), and the interaction between marker SNPs and environments (GE); and between families and environments (FE). (M1: E + L + G; M2: E + L + G + GE; and M3: E + L + G + GE + FE).

Figure 3 depicts the mean (five replicates randomly selected) average correlation between predicted and observed values across the nine environments for different training set sample sizes and compositions consisting of NO-RILs and O-RILs, and three prediction models (M1: E + L + G [gray color]; M2: E + L + G + GE [blue color]; and M3: E + L + G + GE + FE [orange color]). The solid thick line represents the mean average correlation corresponding to the largest training set size (195 RILs) while the thin dashed lines correspond to the reduced training set sizes (185–95). The starting point on the left side of the lines represents the mean average for the scenarios where all the genotypes were observed only once across environments (195, 185, … , 95), while the other extreme of the lines (right side) corresponds to the case where most of the genotypes (e.g., 190 out 195; 170 out 175, … , 90 out of 95) are common across environments. Hence, the number of common genotypes increases as it moves laterally (left to right) across Figure 3.

The highest mean average correlation was obtained with model M3 (∼0.57), which was roughly 33% and 90% higher than M2 (∼0.40) and M1 (∼0.30), respectively (Figure 3). A slight improvement in predictive ability was observed by increasing the number of O-RILs by 10, reaching a plateau with the addition of more O-RILs, followed by a slight reduction in the average correlation towards the end (right side). Models M2 and M3 were less influenced by increasing the number of common genotypes across environments as compared to model M1 (Figure 3). In addition, as expected, the reduction of the training set size resulted in a reduction of the mean average correlation in all three models, being more pronounced in model M3. However, M3 always returned the best results for the same combinations between the training set size and calibration composition compared to M1 and M2 (Figure 3).

Considering the largest training set size (195 RILs), the training composition did not have a significant impact on the model performance, particularly in model M3 which performance was stable across all training compositions. Consequently, model M3 has advantages since high predictive ability does not require testing a large set of common RILs across environments which could be challenging due to constraints on the land and seed availability, especially in the earlier stages of the soybean breeding pipeline.

#### 3.2.2 Within ancestry groups

Since the RILs were derived from families with different groups of ancestry (G1, G2, and G3), the effects of ancestry groups on the predictive ability were assessed by computing the correlation between predicted and observed values of similar families within each environment. For this, a post-stratification of the vector of predicted values of size 1,560 in the three different groups was conducted. Then, the within environments predictive ability for each group of families were computed despite the training set composition.

**FIGURE 4:**
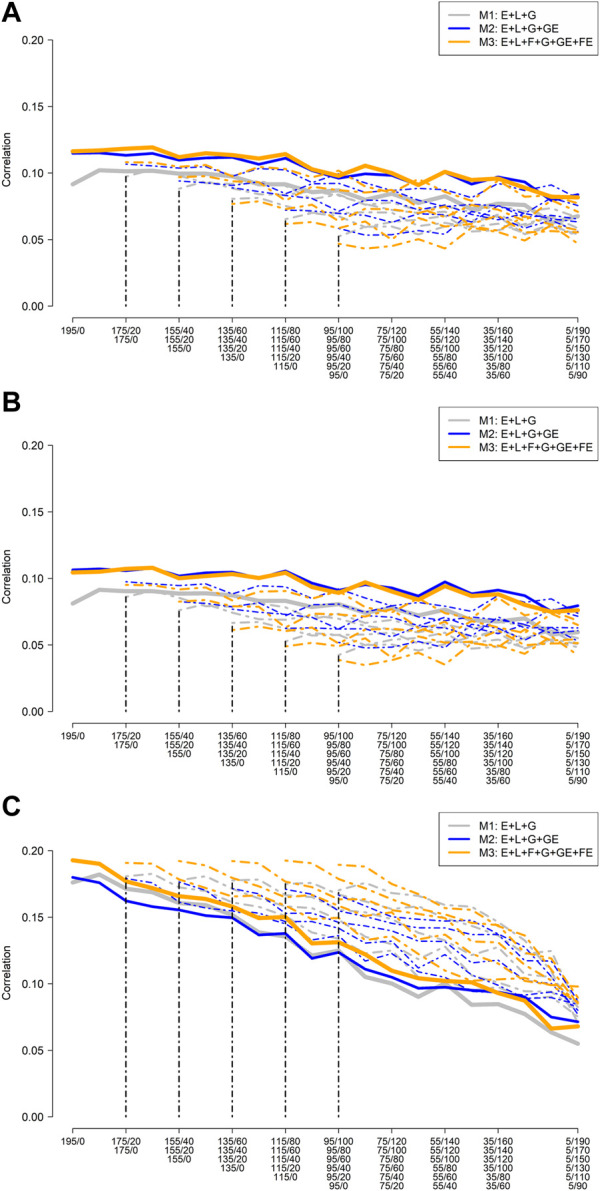
Mean (five replicates) average (across nine environments) of the within environments correlation between predicted and observed values for different genetic backgrounds [**(A)** G1: 17 high-yielding lines, **(B)** G2: 15 diverse ancestries, and **(C)** G3: eight exotic plant introductions (PI)], sample sizes and composition for model training and three prediction models (M1: E + L + G; M2: E + L + G + GE; and M3: E + L + G + GE + FE).

Similar to Figures 3, 4 depict the mean average correlation across environments and replicate computing the correlation between predicted and observed values only among the families that belong to the same group of ancestry (e.g., G1, G2, and G3). As expected, different patterns were observed for the different groups of families. The group of RILs derived from elite parents (G1) was highly affected by the reduction of the training sample size (Figure 4). However, these results significantly improved when the number of O-RILs increased. Using the largest training set size, the best results were obtained with the model that includes the interaction between families and environments (M3). Also, the predictive ability was not affected by the different training set compositions in this case. In the group of RILs derived from diverse ancestry (G2), a less pronounced decay in predictive ability was observed when the training set size was decreased (Figure 4). The best results were also obtained with the most complex model M3. For the group of RILs derived from exotic ancestry (G3), the results using the most complex model were not affected by the training set size and composition (Figure 4). In this scenario, even the smaller training set size returned comparable results to the largest training set size.

#### 3.2.3 Within families for each ancestry group

A more detailed dissection of the model’s predictive ability at the group/family (G1/families 1–17; G2/families 18–31; and G3/families 32–39) level is displayed in Supplementary Figure S3 (G1), Supplementary Figure S4 (G2), and Supplementary Figure S5 (G3) for the different models (M1-M3), training set sizes and composition between overlapped and not overlapped RILs. For each family the within environment correlation between predicted and observed values was computed. Then, the average across environments was obtained for each one of the five replicates and the mean was calculated. This procedure was repeated for all the different training set size and compositions and the results were grouped (mean) for each group of families (G1, G2, and G3).

Different patterns were observed for each one of the ancestry groups. For the G1 group (Supplementary Figure S3), the mean average within families correlation varied between 0.05 and 0.114. Considering the largest training set size, the models M2 and M3 performed very similarly across the different training compositions (blue and orange thick lines) always outperforming M1. However, reducing the training set size, the M3 model loses predictive ability compared to M2. With respect to the families in G2, the mean average within familie correlation ranged from 0.06 to 0.145. When considering the largest training set size, the M3 model slightly outperformed M2 model, specifically with a low number of overlapping RILs. While with reduced training set sizes and compotions not clear pattern were observed. Regarding the families in G3, the correlations varied between 0.55 and 0.195. In this case, the M3 model returned the best results for almost all training compostions when considering the largest training set size. Also, the model’s predictive ability was not significantly affected by reducing the training set sizes but for the training composition when increasing the number of overlapped RILs across environments. Although for each group of families different patterns were observed, in general, the model M3 returned comparable results to the other models (M1 and M2) for most of the training set sizes and compositions. In many of these cases, the M3 model outperformed M1 and M2 specially when considering larger training set sizes and a low number of overlapping RILs across environments.

#### 3.2.4 Maximized (SSD.max) and minimized (SSD.min) genetic diversity

Besides the method that randomly selects RILs to compose the training set, the SSD algorithm that chooses RILs maximizing/minimizing the genetic diversity (SSD.max and SSD.min, respectively) for a fixed sample size that was also considered. Supplementary Figure S6 shows the progression of $E\left(S^{2}\right)$ for maximizing (SSD.max left panel) and minimizing (SSD.min right panel) the genetic diversity contained in a sample of size 195 RILs. A total of 183 iterations were required in SSD.max to meet the stopping criteria when the $E\left(S^{2}\right)$ of the (*k+1*)^th^ iteration is larger than the previous one. On the other hand, a total of 182 iterations were needed to meet the stopping criteria in SSD.min. The corresponding $E\left(S^{2}\right)$ values that maximize/minimize the genomic diversity were 896 and 5,613.

**FIGURE 5:**
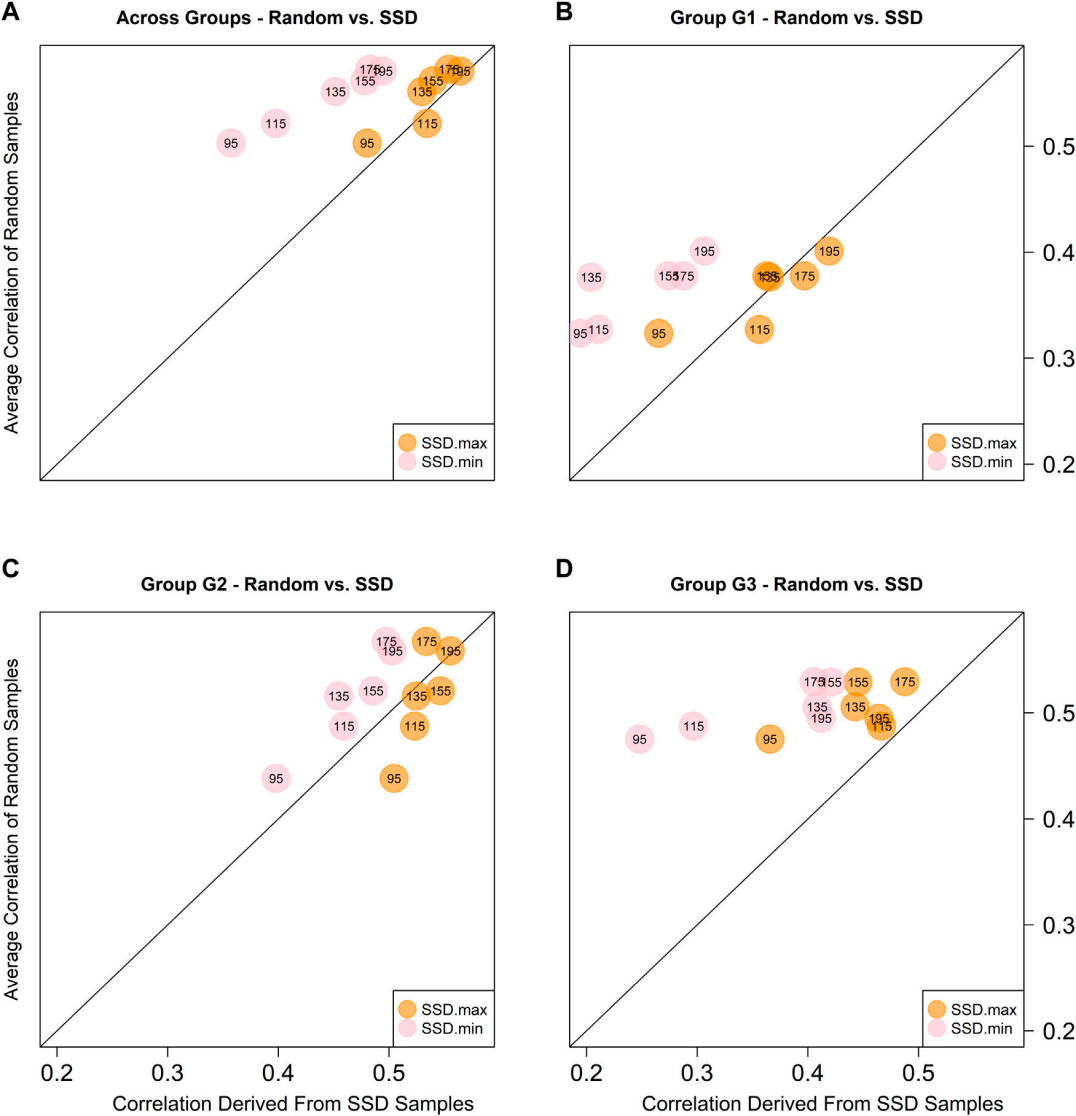
Predictive abilities between random and SSD methods considering sets of common genotypes across environments using model M3 across ancestry groups **(A)**, and within ancestry groups (G1: **(B)**, G2: **(C)**, and G3: **(D)**. The numbers within the circles indicate the sample size of the training set. The orange circles contrast the results obtained with the sample that maximizes the genetic diversity (SSD.max) vs. the random samples. The pink circles contrast the results obtained with the sample that minimizes the genetic diversity (SSD.min) vs. the random samples.

Given the highest prediction accuracies were always obtained with model M3 for all training set sizes, the predictive ability comparison between the random samples and those obtained using the SSD method was based on this model only. Figure 5 depicts the scatter plot between the across environments average predictive ability corresponding to the five random samples (considering only the sets of common genotypes) and the SSD selection method across ancestry groups (panel A) and within ancestry groups (G1-panel B; G2-panel C; and G3-panel D). The colored circles indicate the samples that are being compared: orange color for contrasting SSD maximizing genetic diversity vs. random sample; and pink color for SSD minimizing genetic diversity vs. random sample. The numbers within the circles indicate the training set size, and the diagonal line represents the 1:1 ratio between both methods. Values above the diagonal line indicate that the random method is superior and *vice versa*. In general, across groups of ancestry, equivalent results between the random and SSD.max were observed for all sample sizes. On the other hand, the random sample method outperformed SSD.min in all groups of ancestry and sample sizes (Figure 5).

## 4 Discussion

The implementation of GP has revolutionized commercial and public soybean breeding programs by allowing plant breeders to predict the phenotype of interest in unobserved genotypes (Jarquin et al., 2014a; Jarquin et al., 2014b; Persa et al., 2020; Widener et al., 2021; Canella Vieira et al., 2022). The first report on GP in soybean was based on a standard G-BLUP model including only additive effects and an extended version of the G-BLUP model including additive-by-additive effects (Jarquin et al., 2014b). Ma et al. (2016) used ridge regression best linear unbiased prediction (rrBLUP) (Endelman, 2011) with fivefold cross-validations to explore strategies of marker preselection. Prediction accuracy based on pre-selected markers slightly increased compared with random or equidistant marker sampling (Ma et al., 2016). Xavier et al. (2016) showed that the training population size was the most impactful factor in the prediction accuracy when investigating the impacts of training population size, genotyping density, and different prediction models. Persa et al. (2020) expanded the reaction norm model proposed by Jarquin et al. (2014a) by incorporating the interaction between the family’s membership of the genotypes and the environment under the premise that the differential responses of families to environmental stimuli could be used for enhancing the selection process in target environments. These authors showed significant improvements in predictive ability by incorporating the family-by-environment interaction in the models compared to the conventional reaction norm model (Persa et al., 2020). However, they also pointed out that this model requires to observe, at least partially, the individuals of the family to predict otherwise no improvements in predictive ability are expected when predicting a yet to observe family. Thus, this model is benefited only when some individuals of the target family are observed. In this case, the family term here has a closely related to pedigree information. In addition, Habier et al. (2007) and (2013), showed that the addition of a model term modeling the differences between groups of indiviuals (families, pedigree, etc.) besides the genomic information, does a better job capturing relationship information rather than just relationships alone. In this way, the family term enables the genomic information to focusing on only capturing LD information rather than trying to capture or explain at the same time LD and the relationships.

Canella Vieira et al. (2022) investigated the potential of incorporating soil texture information and its interaction with molecular markers via covariance structures for enhancing predictive ability across breeding scenarios. The obtained results were more stable when predicting trait performance in novel environments compared to the conventional reaction norm model.

The expression of a phenotype is a function of the genotype, the environment, and the interaction between the genotype and environment (G×E) across different environments (de Leon et al., 2016). Grain yield is a highly complex and quantitative trait regulated by numerous large and small-effect genes, of which its expression largely depent on the genotype interaction with various components of the environment. Genomic prediction-based sparse testing is a promising concept to substantially increase the number of tested genotypes and environments while maintaining fixed requirements for land, seed availability, and costs (Jarquin et al., 2020b; Montesinos Lopez et al., 2023a). Hence, GP-based sparse testing enables a more comprehensive assessment of genotype stability and adaptation in the early stages of the breeding pipeline, which likely could result in a more accurate selection of superior genotypes widely adapted to various environments.

In soybean breeding, this is particularly important in the early stages of yield trials (i.e., preliminary yield trials) where seed availability and the excessive number of testing genotypes are major constraints (Canella Vieira and Chen, 2021). In the public sector, preliminary yield trials often consist of thousands (∼1,000 to 3,000) of genotypes tested across several replicated environments (three to seven environments, two to three replications) resulting on average 20,000 preliminary yield plots (2,000 genotypes, five environments, two replications). As shown in this research (1,755 genotypes, nine environments, a total of 15,795 phenotypic records), GP-based sparse testing can sustain high accuracies at various training compositions which could reduce the testing footprint by as much as 90% (Figure 1). Given the cost of approximately $15 for a yield phenotype (plot), this methodology has the potential to reduce the total cost of preliminary trials (depending on the size of the program) by as much as $210,600 ($236,925–26,325; 1,755 vs 15,795) with the largest training set size (195) while about $224,100 ($236,925-$12,825; 855 vs 15,795) for the smallest one (95) while maintaining the number of genotypes and environments fixed. On the other hand, the number of tested RILs and/or environments can increase by 5- to 10-fold while maintaining the costs fixed. Considering the premise of same phenotyping cost across environments (fixed cost of $15 per phenotypic record), with the initial budget dedicated to test all the RILs in all of the environments ($236,925) the number of RILs or the number of environments can be increased by 9 folds or considering combinations of these.

In this study, different prediction models, as well as multiple training set sizes and compositions (ratio of NO-RILs and O-RILs across environments, SSD.max and SSD.min), were investigated. The most comprehensive model, including the interaction between families and the environment (M3), yielded the highest prediction accuracies independent of training set sizes and composition. The ability to borrow information between genotypes derived from the same families likely contributed to the superior performance of M3 because half of the cross was already observed a considerable number of times in other crosses and environments (Jarquin et al., 2020a). In GP-based sparse testing where most genotypes are untested within and across environments, borrowing information from tested individuals within the same family but observed in different environments has been shown to improve predictive ability by as much as 48% compared to models including the interaction of molecular markers and environments only or reaction norm model (Persa et al., 2020). Across all three models, reducing the training set size negatively impacted prediction accuracies. However, prediction accuracy was rapidly recovered by the addition of O-RILs. This indicates that the training set composition is critical to a successful GP-based sparse testing implementation when the training set is reduced. This observation is also important in advanced yield trials where the number of testing genotypes is significantly reduced as compared to preliminary trials (roughly 90% fewer genotypes), and the number of testing environments is significantly increased (Canella Vieira and Chen, 2021). Specifically, the advanced yield trials stage offers more flexibility in maximizing training set sizes and composition (increasing O-RILs) since seed availability should not be a major constraint.

The results and trends in predictive ability obtained with models M1 (main effects only) and M2 (reaction norm considering the interaction between markers and environments) were similar to those obtained by Jarquin et al., 2020b; Crespo-Herrera et al., 2021 analyzing maize and wheat data, respectively. However, these authors did not consider models including the interaction between family and environments. On the other hand, the results of model M3 (also including the interaction between family and environment) were similar to those obtained by Persa et al., 2020 analyzing information of the SoyNAM experiment comprising 1,358 RILs observed in 18 environments (not all RILs observed in all environments) but considering a conventional fivefold cross-validation predicting tested genotypes in observed environments (CV2) and untested genotypes in observed environments (CV1). In addition, Xavier and Habier (2022), using the SoyNAM population conducted an study to evaluate the effects in predictive ability of models similar to M1 and M2 when considering simulated data for the case where RILs are observed only once across environments for different heritabilities. The results obtained for these authors were higher compared with the results here presented probably due to the fact they considered simulated data as response as opposed to real data and a different cross-validation scheme.

In this study, in addition to the ratio of NO-RILs and O-RILs, the selection of RILs to compose the training set based on maximizing (SSD.max) and minimizing (SSD.min) genetic diversity was also investigated. The Super Saturated Design (SSD) methodology was implemented to identify the set of 195 RILs that either decreased or increased E(*S* ^2^) (SSD.max and SSD.min, respectively). The method successfully created two fixed-size sample groups with contrasting genetic diversity. This was further observed in the population structure of each group, where SSD.max samples showed wider distribution and minimal clustering as compared to the SSD.min samples (right panel in Figure 2). Interestingly, the SSD.max samples' prediction accuracy outperformed the SSD.min samples in all scenarios (Figure 5). Superior prediction accuracy in the SSD.max samples could be explained by the higher availability of diverse alleles associated with stress resilience, and therefore, resulted in higher stability across environments. A narrower genetic diversity, although may prioritize high-yielding alleles, can be more susceptible to environmental stressors. This was also observed in the higher phenotypic correlation of the SSD.max samples across all environments compared to SSD.min samples (Supplementary Figure S2).

Also, in general the results of the SSD.max sample slightly under performed the results of the random samples for most of the sample sizes of common RILs across environments and across ancestry groups, except for the case when the sample size was fixed in 115 common RILs (Figure 5A). Considering the different ancestry groups, mixed results were obtained for G1, while for G2 better results were obtained with the SSD.max sample, and for G3 the random samples were superior. In all of the cases, the SSD.min returned the worse results. Seems like the G1 group of families (high yielding) is very susceptible to the presence of genotype-by-environment interactions thus reduced sample sizes with a high propotion of non-overlapped genotypes significantly reduced predictive ability. On the other hand, in G2, the reduction of the training set sizes is the only factor that reduces predictive ability, and the results are more stable across the different training sets compositions. Finally, for G3, neither the sample size nor the training set composition seems to affect the predictive ability since similar results were obtained across these.

Additionally, higher genetic diversity (here represented by G3) yielded stable prediction accuracies across various training set sizes and compositions. Like the SSD.max observation, this could be attributed to the higher availability of diverse stress resilient-alleles and therefore require a smaller number of samples, as well as reduced O-RILs to achieve maximum prediction accuracy. These observations are fundamental to the establishment of a successful GP-based sparse testing design and should be further explored across populations with various genetic backgrounds, including high-yielding bi-parental populations.

Similarly to the analysis of the different genetic backgrounds, a more detailed analysis can be done by considering the model’s predictive ability at the family level. As expected, a significant reduction in predictive ability was obtained (Supplementary Figures S4–S6); however, there was not a unique model always outperforming the other two. In general, model M3 slightly outperformed the other two models, specially when considering the largest training set size and with a lower number of overlapping genotypes. Also, it was superior when only considering the families from G3. Arguably the results obtained when computing the correlation across families might be inflated; however, the selection of the superior cultivars are not made within families but across these. In addition, considering the three models, there was not a unique model systematically outperforming the others. The lack of a unique model significanly outperforming the within families predictive ability is due to the fact that no relationship is factored in when ranking individuals within families (Habier et al., 2013). All the models have the same ability to model LD. In our case, at the family level, the most complex model M3 showed slight improvements in predictive ability by including the family term as main effect and in interaction with environments. This could help breeders to better screening RILs across families and environments while reducing phenotyping cost via sparse testing designs.

## 5 Conclusion

Genomic prediction-based sparse testing design is a promising approach to further maximize the applications of high-dimensional genomic data and predictive models toward improving cultivar development. The increase of testing environments in the early stages of the breeding pipeline can provide a more comprehensive assessment of genotype stability and adaptation which are fundamental for the precise selection of superior genotypes widely adapted to various environments. Various training set sizes and compositions, as well as prediction models, have been investigated. Overall, the training set size and the inclusion of O-RILs appear to be the main factors impacting prediction accuracy given a fixed training set size while the genetic diversity seems to be a secondary factor, except when it was minimized returning the worse results. Additional studies investigating the real-world effectiveness of prediction accuracy based on genotype ranking and advancement breeding decisions can help determine the ideal protocols for GP-based sparse testing in soybean. In summary, GP-based sparse testing can either improve/increase testing capacity (represented as the number of genotypes and environments) at a fixed cost or substantially decrease the cost of a breeding pipeline at a fixed testing capacity.

## Data Availability

Publicly available datasets were analyzed in this study. This data can be found here: https://soybase.org/SoyNAM/.
