# Supplementary material for: Improving predictive ability in sparse testing designs in soybean populations
Source: Front Genet. 2023 Nov 23;14:1269255. doi: 10.3389/fgene.2023.1269255 (PMC10701390; doi:10.3389/fgene.2023.1269255)
Supplement: Supplementary file 1 [file DataSheet1.docx]

# Supplementary Material


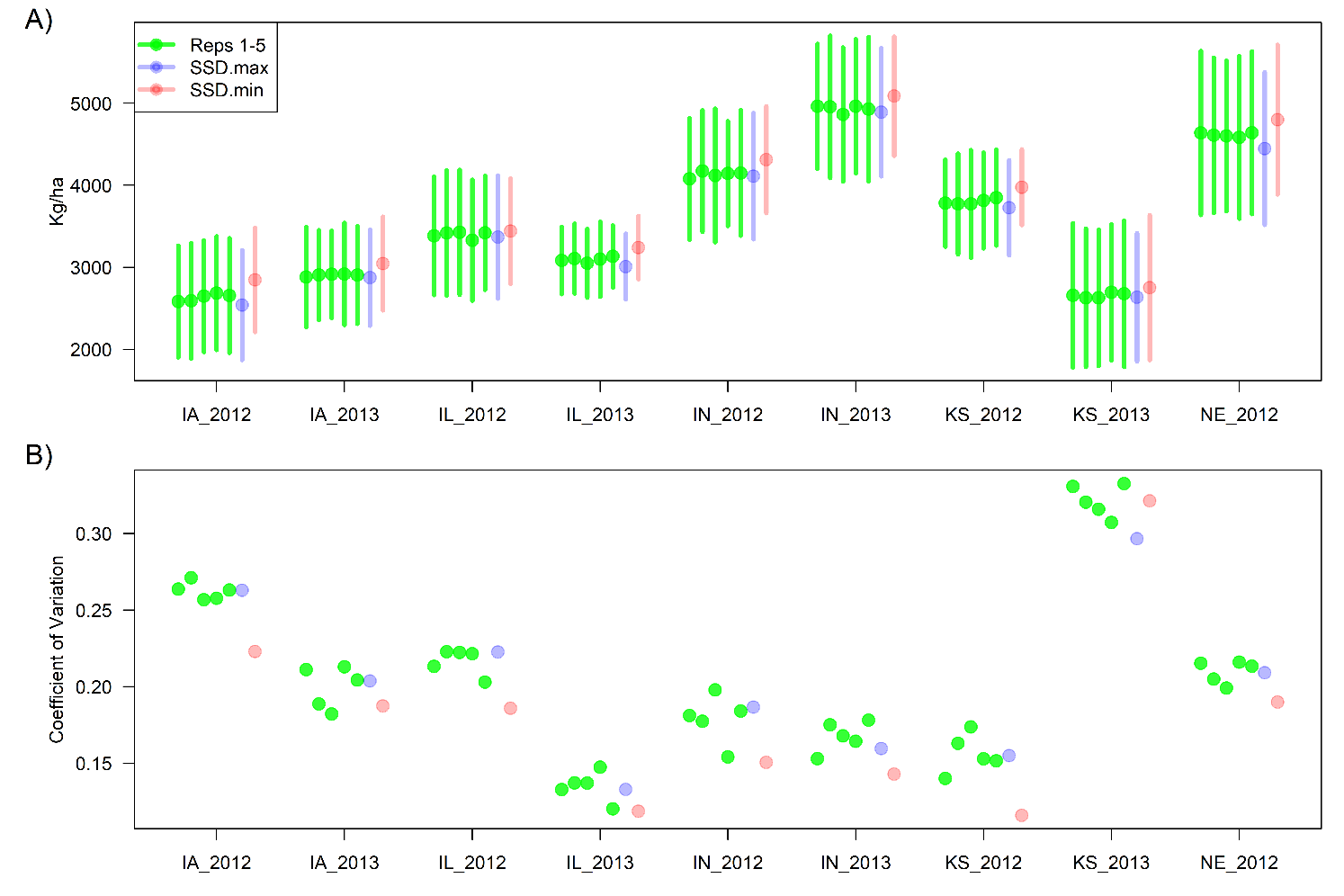
 **Figure S1.**  A) Yield means (kg ha^-1^, ± corresponding standard deviations) of selected samples for each environment. Samples included five randomly selected samples where each genotype is observed only once across environments and two samples of 195 NO-RILs based on the methods for maximizing/minimizing the genetic diversity. B) Coefficients of variation of selected samples for each environment.


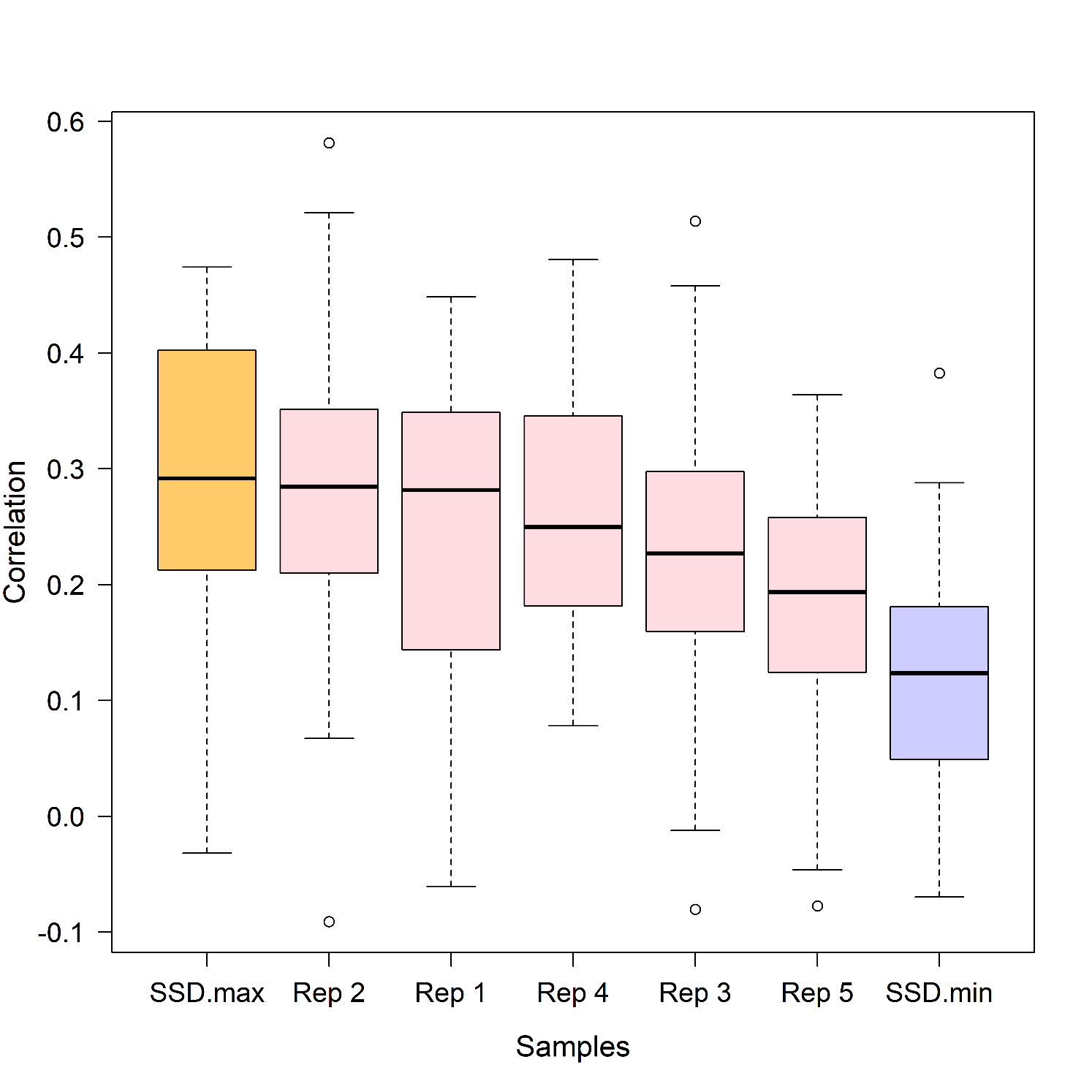
 **Figure S2**. Boxplot of the phenotypic correlation among the nine environments $\left( \frac{9\left( 9-1 \right)}{2}=36 \mathrm{pairs} \right)$ for each one of the seven samples. The samples are ordered from the highest to the lowest based on the mode of their corresponding correlations.


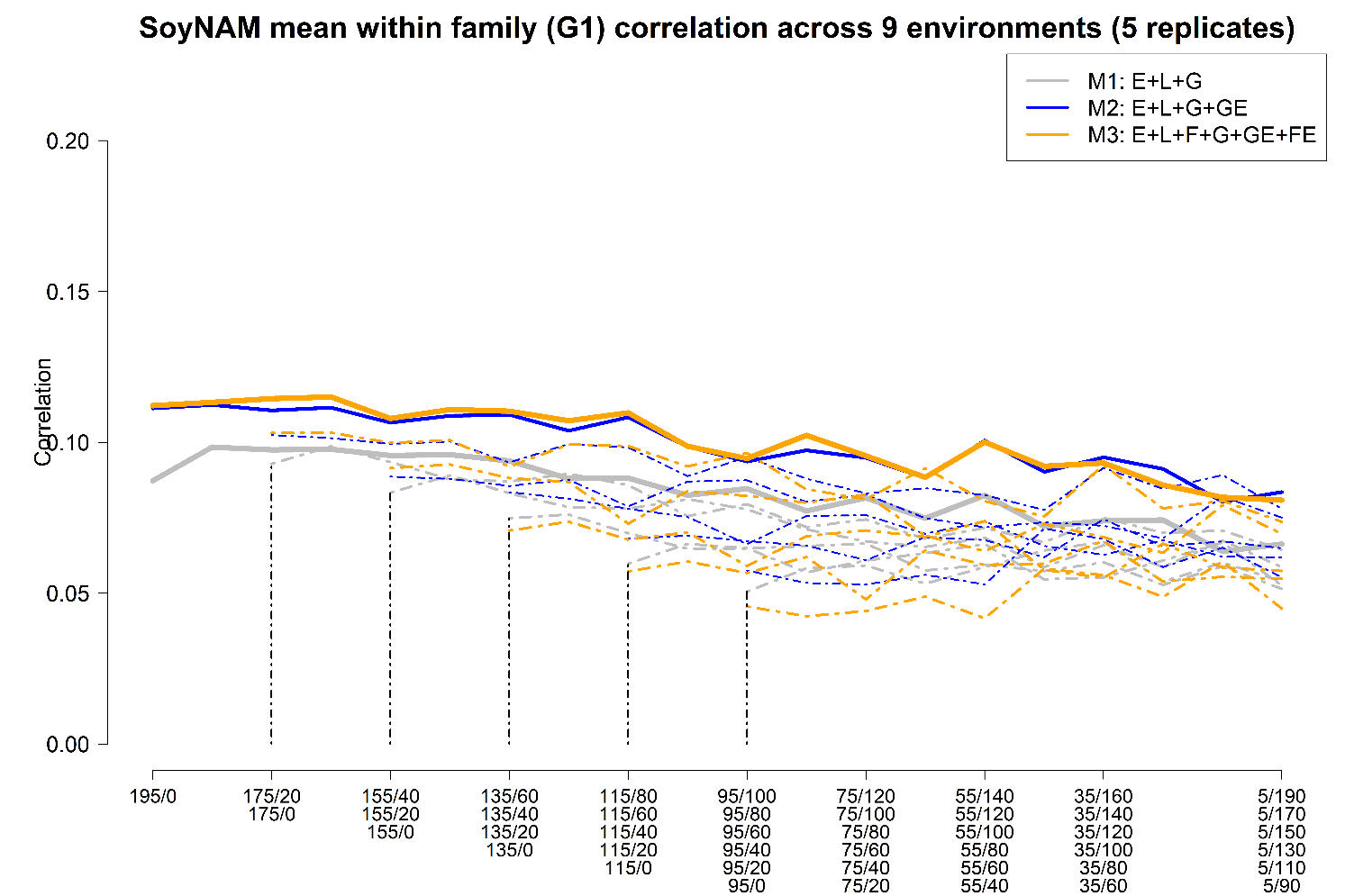


**Figure S3**. Mean (five replicates) average (across nine environments) of the within environments correlation between predicted and observed values for the families (1-17) corresponding to group G1, for different sample sizes and composition for model training and three prediction models (M1: E+L+G; M2: E+L+G+GE; and M3: E+L+F+G+GE+FE).


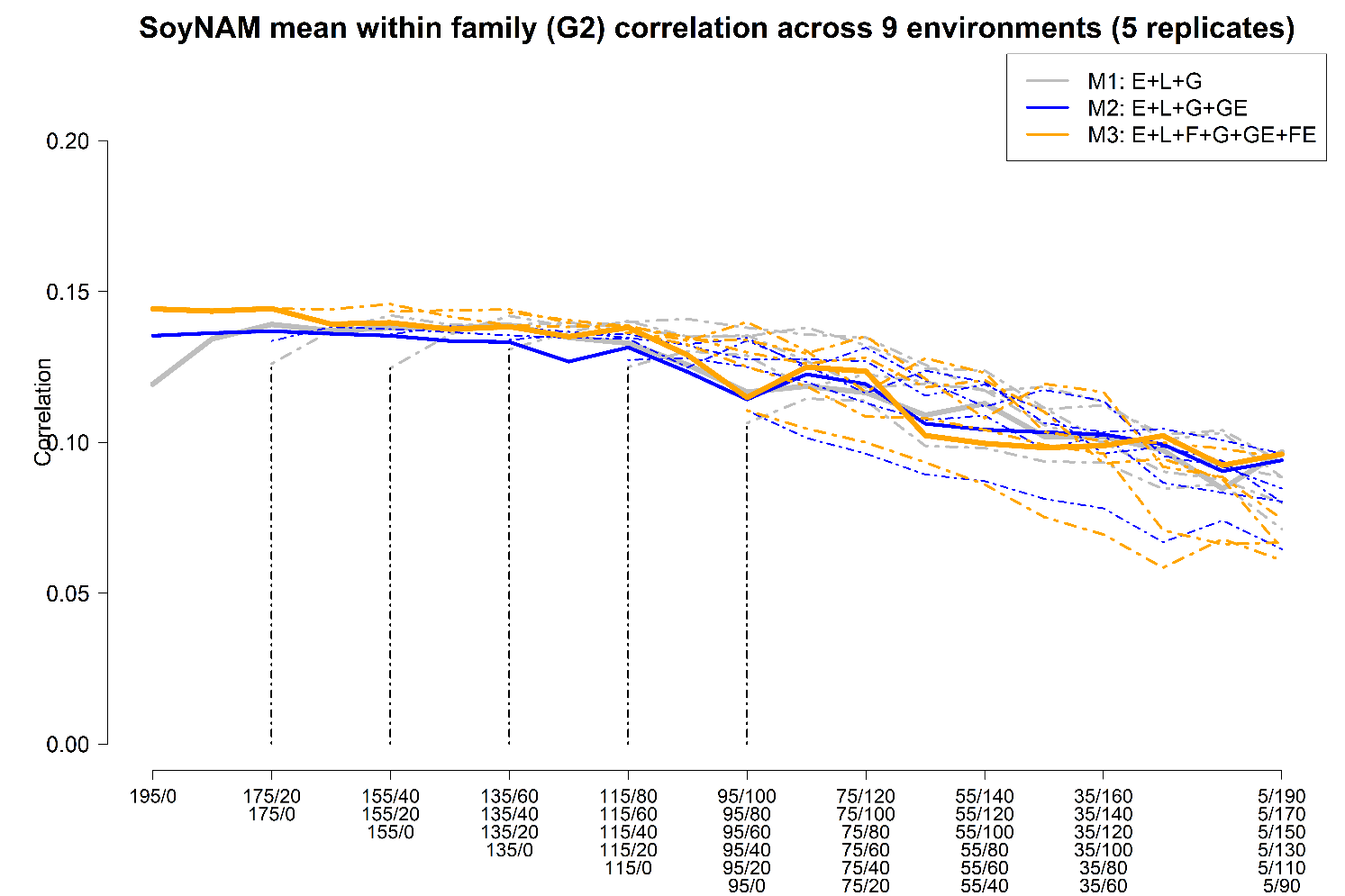


**Figure S4**. Mean (five replicates) average (across nine environments) of the within environments correlation between predicted and observed values for the families (18-31) corresponding to group G2, for different sample sizes and composition for model training and three prediction models (M1: E+L+G; M2: E+L+G+GE; and M3: E+L+F+G+GE+FE).


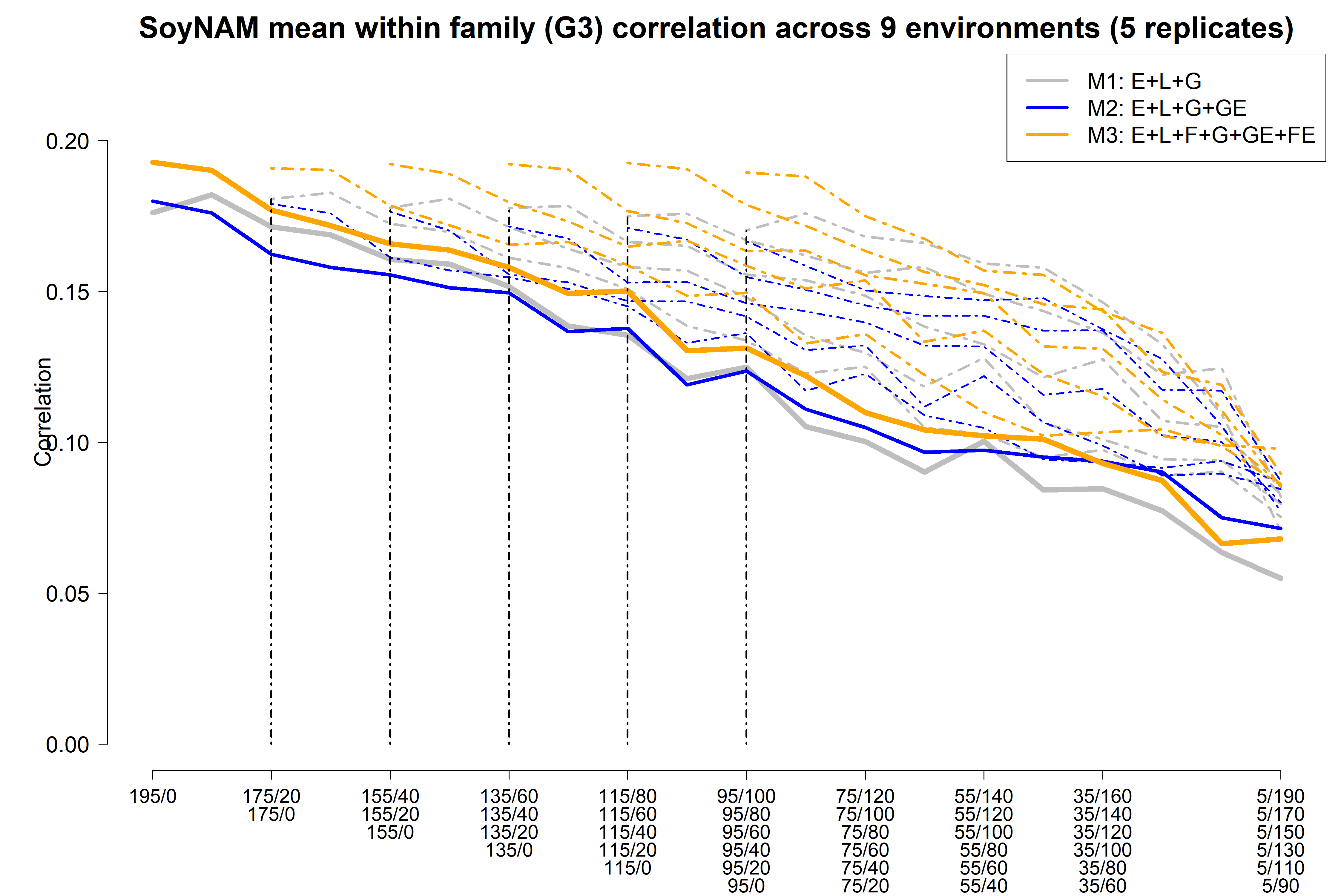


**Figure S5**. Mean (five replicates) average (across nine environments) of the within environments correlation between predicted and observed values for the families (32-39) corresponding to group G3, for different sample sizes and composition for model training and three prediction models (M1: E+L+G; M2: E+L+G+GE; and M3: E+L+F+G+GE+FE).


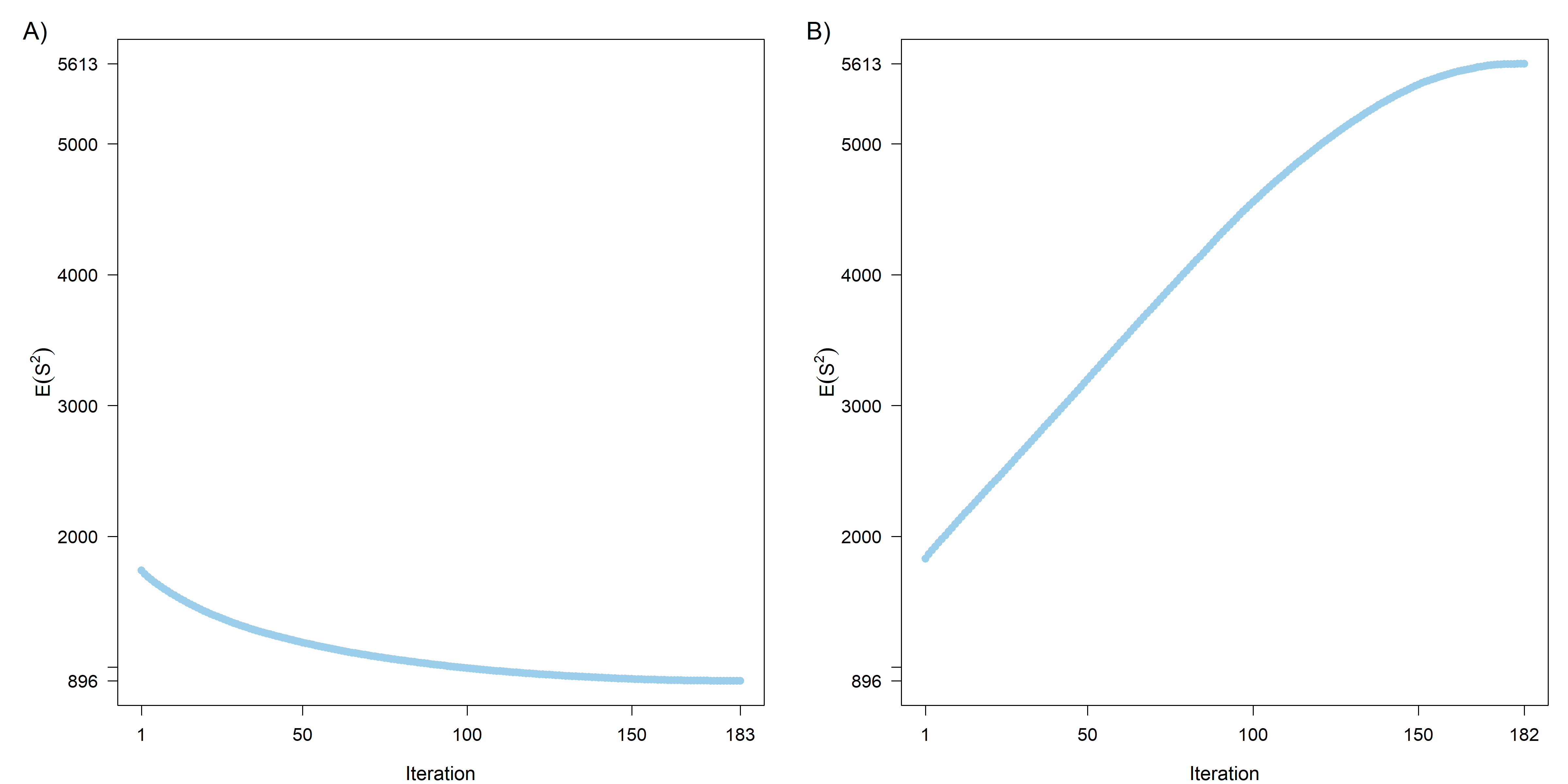
**Figure S6**. Progression of the $E\left( S^{2} \right)$ for increasing (left panel, SSD.max) and decreasing (right panel, SSD.min) the genetic diversity for a sample of 195 RILs out of the 1,755 RILs. Low values of the $E\left( S^{2} \right)$ return samples with maximized genetic diversity and *vice versa*.
